# Serial Dependence in Dermatological Judgments

**DOI:** 10.3390/diagnostics13101775

**Published:** 2023-05-17

**Authors:** Zhihang Ren, Xinyu Li, Dana Pietralla, Mauro Manassi, David Whitney

**Affiliations:** 1 Vision Science Graduate Group, University of California, Berkeley, Berkeley, CA 94720, USA; 2 Department of Psychology, University of California, Berkeley, Berkeley, CA 94720, USA; 3 Institute of Sociology and Social Psychology, University of Cologne, Albertus-Magnus-Platz, D-50923 Cologne, Germany; 4 School of Psychology, King’s College, University of Aberdeen, Aberdeen AB24 3FX, UK; 5 Helen Wills Neuroscience Institute, University of California, Berkeley, Berkeley, CA 94720, USA

**Keywords:** serial dependence, semantic similarity, computer vision, medical image perception, skin cancer diagnostic, systematic error

## Abstract

Serial Dependence is a ubiquitous visual phenomenon in which sequentially viewed images appear more similar than they actually are, thus facilitating an efficient and stable perceptual experience in human observers. Although serial dependence is adaptive and beneficial in the naturally autocorrelated visual world, a smoothing perceptual experience, it might turn maladaptive in artificial circumstances, such as medical image perception tasks, where visual stimuli are randomly sequenced. Here, we analyzed 758,139 skin cancer diagnostic records from an online app, and we quantified the semantic similarity between sequential dermatology images using a computer vision model as well as human raters. We then tested whether serial dependence in perception occurs in dermatological judgments as a function of image similarity. We found significant serial dependence in perceptual discrimination judgments of lesion malignancy. Moreover, the serial dependence was tuned to the similarity in the images, and it decayed over time. The results indicate that relatively realistic store-and-forward dermatology judgments may be biased by serial dependence. These findings help in understanding one potential source of systematic bias and errors in medical image perception tasks and hint at useful approaches that could alleviate the errors due to serial dependence.

## 1. Introduction

The natural visual world is autocorrelated: objects do not spontaneously pop into or out of existence in the typical visual experience, and what was present a moment ago tends to still be present at this moment. The human visual system has developed adaptive mechanisms that take advantage of these natural autocorrelations by introducing serial dependence in perceptual interpretations. Due to this mechanism, objects recognized at one moment appear more like similar objects seen in the last several seconds. The result of this serial dependence is that perceptual experience seems smoother and more stable than it should be. This is beneficial because without it, the visual world would look jittery and unstable; object identities would appear to fluctuate due to changes in lighting, viewpoint, blinks, and myriad sources of internal and external noise [1,2].

It is intuitive that human vision benefits from recycling visual history, smoothing and stabilizing perceptual experience in the natural world. However, the benefit of serial dependence has limits because the visual world is not always natural. In certain artificial, human-designed visual tasks, such as medical image perception or randomized laboratory experiments, visual stimuli are no longer naturally autocorrelated. Visual images in these situations can vary randomly from one moment to the next. If the visual system imposes serial dependence, smoothing or reusing previous visual history, this could introduce systematic errors by attracting current perception towards previous visual history.

Studies have shown that this is exactly what happens. Serial dependence systematically biases current perception toward visual history in many tasks, such as perception of orientation [1], attractiveness [3,4], and emotional expression [5]. Serial dependence also introduces systematic perceptual errors in medical image perception tasks [6]. However, these studies were conducted under lab conditions with highly artificial stimuli and experimental designs that are not typical in clinical practice [6]. More recently, progress in Generative Adversarial Networks (GANs) affords the opportunity to generate more realistic simulated medical images as stimuli [7,8]. However, even in these studies, the relatively complex psychophysical tasks were not comparable to realistic, clinically relevant scenarios.

In this study, we address several of the shortcomings in prior work and we test whether serial dependence occurs in a teledermatology setting, one of the most important and commonly employed subsets of telemedicine [9,10]. Remote store-and-forward teledermatology, which involves sequential judgments of static images, is an especially fast growing area of telemedicine [11,12,13,14], and it requires the involvement of clinicians because automated systems are not sufficient to make accurate diagnostic classifications [15,16,17]. The question in the present study is whether sequential judgments of dermatological lesions in a remote store-and-forward setting result in serial dependence.

We analyzed 758,139 skin cancer diagnostic judgments from 1137 participants collected from an app developed by Centaur Labs, a US medical Artificial Intelligence (AI) company based in Boston. The task was a straightforward 2AFC (two-alternative forced choice) (yes/no) discrimination, with a goal of diagnosing whether an actual skin cancer image was nevus (benign) or melanoma (malignant). This is comparable to a realistic, remote store-and-forward teledermatology task, with a more natural two-alternative forced choice (yes/no) design.

We found that there was statistically significant serial dependence in discrimination judgments that was tuned to the sequential similarity in the malignancy of the lesions. The consequence of the serial dependence was a statistically significant reduction in metrics of sensitivity and specificity, including reduced d-prime (d′) and increased error rates. Additionally, using a recent Learned Perceptual Image Patch Similarity (LPIPS) computer vision model, we quantified serial dependence as a function of the semantic similarity between sequential images and found that serial dependence varied as a function of the patchwise similarity between sequential images.

Together, our results suggest that serial dependence in perceptual decisions may impact realistic dermatological judgments, at least under certain circumstances akin to those in remote store-and-forward teledermatology [18,19].

## 2. Materials and Methods

### 2.1. Experiment Stimuli

**Figure 1:**
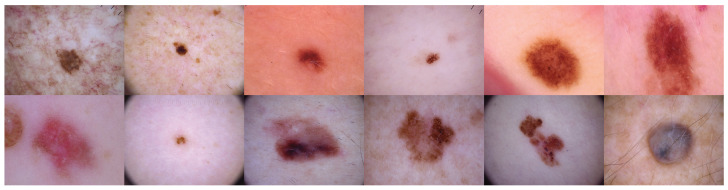
Samples of skin cancer image stimuli. A total of 7798 images were drawn from the ISIC 2019 Challenge Datasets [20,21,22], which contain various nevus and melanoma lesions. In each trial, a single random sample image was selected and presented to the participant. Observers judged whether the image was nevus (benign) or malignant (yes/no forced choice design). Feedback was provided after each trial.

All skin cancer images utilized in the trials on the app were subsampled from ISIC 2019 Challenge Datasets [20,21,22]. This set of images contains two types of lesion, i.e., nevus and melanoma, indicating benign and malignant cases. The images were dermoscopy images after manual correction of color hue, luminance, and alignment and were taken by different devices using polarized and non-polarized dermatoscopy. Samples of skin cancer image stimuli are shown in Figure 1. In summary, for all the skin cancer images that were shown, 57.3% were benign and 42.7% were malignant.

### 2.2. Participants

The users of the app are predominantly medical students, with some medical residents. Individual participant information such as age, sex, and demographics that are typically gathered in scientific experiments are not known for this group of observers because this information is saved in the user profile of the app and was not available to us. However, it is known that all users had normal or corrected-to-normal vision. Since the use of the app does not work outside of the United States, users must be located in the U.S. at the time of app usage. Before using the app, users gave consent to have Centaur Labs use the data they provide through app usage. Users received earnings from a predefined money pool (around US$ 50) for each task they participated in.

### 2.3. Experiment Design

For the dermatological classification task that was investigated in this study, users first completed a training session of 10 trials with 10 separate stimuli. This training explained the procedure of the task and prepared users for the actual classification task, which was identical to the training.

In each trial, a random skin cancer image was selected and presented to the participant. Below the image, they were prompted to choose one of the two possible responses, “benign” or “malignant”. Feedback was provided after every trial to inform users if their response was correct or incorrect. Afterward, users voluntarily moved on to the next trial at their own pace. Users were told they could end the task at any time.

We were provided with 758,139 data points across 13 variables, which were collected between 4 September 2020 and 21 June 2021. Each data point corresponded to one decision of a user, classifying a dermatological image as either benign or malignant. After pre-processing, 756,001 data points from 1137 users were used for further analyses (pre-processing steps and exclusion criteria are illustrated in Appendix A).

### 2.4. Serial Dependence

Serial Dependence has three main kinds of tuning. First, feature tuning: serial dependence occurs most strongly between relatively similar features and not between identical ones or highly dissimilar ones [1,23]. For example, when two identical images are seen in succession, serial dependence does not bias judgments in any direction because the images are identical; likewise, if the two successive images are extremely different from each other (e.g., apples and oranges), then serial dependence does not bias judgments either. Only when two successive images are moderately similar is there a serial dependence in perceptual judgments. Serial dependence is also temporally tuned: the magnitude of serial dependence gradually decays over time or with intervening visual information [1,24]. Third, spatial tuning: serial dependence occurs only within a limited spatial region, and it is strongest when previous and current objects are presented at the same location [1,25]. In general, we can utilize feature and temporal tuning as the most important metrics to probe the serial dependence effect and to rule out other artifacts, such as simply repeating the same response or lapsing.

**Figure 2:**
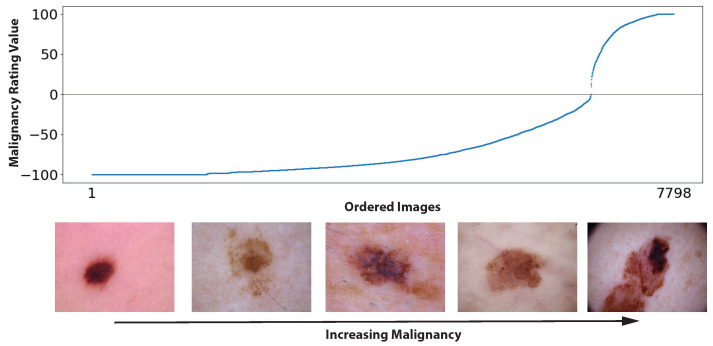
Overview of all 7798 (6688 benign, 1110 malignant) unique images used, sorted by the consensus malignancy rating value (−100: classified as benign by all users, 100: classified as malignant by all users). The five sample images below the abscissa show a sequence of example images that had varying degrees of agreement, from benign to malignant.

To measure the presence of feature tuning, we measured serial dependence as a function of the similarity in sequential stimuli. In this study, we adopt two metrics of similarity. One is malignancy similarity, where malignancy is estimated based on a popularity vote. The “similarity” in this respect is an abstracted concept based on behavioral judgments of independent observers. What counts as similar is not necessarily in the image or pixel domain but in the degree of malignancy (Figure 2). The second form of “similarity” that we quantified is semantic similarity, using a popular Learned Perceptual Image Patch Similarity (LPIPS) metric [26] approach borrowed from computer vision.

### 2.5. Malignancy Similarity

The malignancy of each stimulus was estimated based on a popularity vote: −100 means all users classified the lesion as benign; 100 means all users classified the lesion as malignant. Figure 2 shows the distribution of malignancy over all stimuli. “Malignancy similarity,” used in subsequent analyses of serial dependence, was computed as the malignancy difference between any two sequential stimuli. Any two adjacent stimuli on the abscissa of Figure 2 have high similarity; conversely, any two distantly separated stimuli have low similarity.

### 2.6. Semantic Similarity

**Figure 3:**
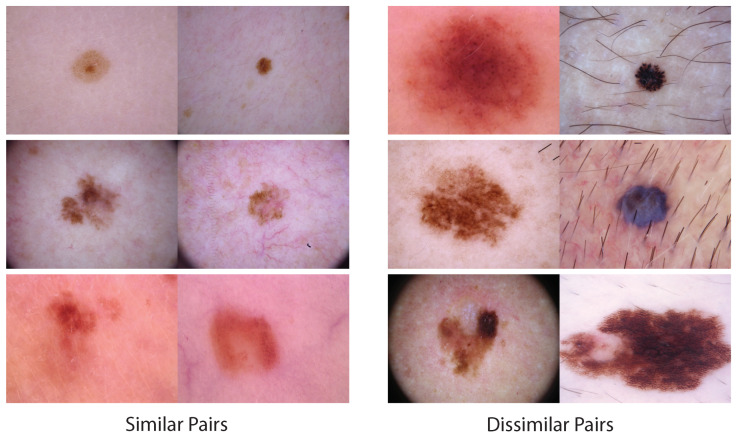
LPIPS semantic similarity [26] example image pairs. Based on this semantic metric, we can group images into similar pairs vs. dissimilar pairs. Note the patch-wise similarity that similar image pairs have.

The semantic similarity is computed via the Learned Perceptual Image Patch Similarity (LPIPS) metric [26]. This is a popular nonlinear similarity metric utilized in computer vision. For deep learning models, there are deep features after each convolutional layer [27,28,29]. The semantic similarity is computed as a sum of weighted differences between the corresponding deep features at different layers. If the semantic similarity is small, two images would share more patch-wise similarity in the pixel domain, with 0 representing identical. In particular, we utilized AlexNet [27] as the backbone of the LPIPS metric. Figure 3 shows two groups of similar and dissimilar skin cancer images based on LPIPS metric. A similar pair is defined as a pair of images whose similarity is less than the mean similarity of all image pairs, and vice versa.

### 2.7. Diagnostic Performance Evaluation

To measure the presence of serial dependence, we analyzed users’ performance in the dermatological classification task. Multiple metrics from signal detection theory were utilized, including Sensitivity or Hit Rate (HR)=TP/(TP+FN), Specificity=TN/(TN+FP), and Error Rate=(FN+FP)/(TP+FN+FP+TN), where “Positive” (P) represents the malignant case, and “Negative” (N) represents the benign case. Then, TP (True Positive), FN (False Negative), FP (False Positive), and TN (True Negative) can be defined accordingly. We also utilized d-prime ($d^{\prime }$) and the criterion (c) to evaluate observers’ discrimination and bias. These can be computed as follows:

$$d^{\prime }=z\left(HR\right)-z\left(FAR\right)$$

c=−0.5∗(z(HR)+z(FAR))

where z(·) is the inverse cumulative distribution function of the standard normal distribution, and False Alarm Rate (FAR)=FP/(FP+TN).

### 2.8. Feature Tuning Analysis

We evaluated the diagnostic performance metrics described above while taking into account the sequential similarity between successive images that each observer saw. There were two types of similarity that we evaluated. In the first one, the malignancy similarity, we computed the n-back similarity as $|M_{t-n}-M_{t}|$, where $M_{t}$ represents the malignancy of the current trial image and $M_{t-n}$ represents the malignancy of the n-back trial image. We used the absolute value of the difference because the sign of the malignancy does not matter. Then, we grouped the malignancy similarities with a group range of 10, resulting in a total of 20 similarity groups. Performance metrics were computed within each group. In the end, we obtained the sensitivity, specificity, d′, c, and error rate in relation to the n-back malignancy similarity.

The n-back semantic similarity can be obtained directly from the LPIPS metric [26], $f(I_{t-n},I_{t})$, where $I_{t}$ represents the current trial image, $I_{t-n}$ represents the n-back trial image, and f(·) is the LPIPS model. Then, the semantic similarities were grouped with a group range of 0.02, with groups that have insufficient trials excluded. We analyzed groups in the semantic similarity range of [0.3,0.68]. Performance metrics were also computed within each group. In the end, we obtained the performance metrics in relation to the n-back semantic similarity.

In order to probe the impact of serial dependence on diagnostic performance, we measured the net change of those metrics relative to what is expected by chance. To conservatively estimate this “chance” baseline, we used the future trial (N + 1) stimulus because this stimulus is not predictable and cannot influence the past. Essentially, because the stimuli are randomly ordered, the current response is only predictive of the future stimulus about half of the time, which gives a baseline estimate of chance performance. If the current judgment is pulled toward the previous stimulus (serial dependence), then the current trial accuracy will decrease relative to that chance performance. By using the future (N + 1) accuracy as a baseline, we control for any systematic response biases that observers might have [30,31]. For example, simply pressing the same button on every trial results in a response bias, but this will not show up as measured serial dependence because the serial dependence is normalized relative to the N + 1 trial.

Finally, we computed the net change in sensitivity, specificity, d′, criterion, and error rate as a function of the sequential similarity between successive images. As serial dependence only occurs for relatively similar features, we expected the serial dependence effect, if present, to be maximal when sequential stimuli are moderately similar.

### 2.9. Temporal Tuning Analysis

After checking the feature tuning characteristics, we fit Gaussian curves (Equation (1)) on top of the net change graphs to quantify the magnitude of the serial dependence effect (as shown in Section 3).

(1) $$f\left(x|\mu ,\sigma^{2}\right)=\frac{a}{\sqrt{2\pi \sigma^{2}}}\;e^{-\frac{{\left(x-\mu \right)}^{2}}{2\sigma^{2}}}$$

where *x* is the data variable, μ and σ are the mean and standard deviation of the Gaussian distribution, and *a* is an amplitude modulation parameter. Here, *a*, μ, and σ will be optimized during curve fitting. After fitting, we report the peak value of the fitted Gaussian curve as the amplitude of the serial dependence effect.

We analyzed the serial dependence effect magnitude of 1-back (N-1), 2-back (N-2), 3-back (N-3), and 4-back (N-4) trials. Then, we obtained the relation between the serial dependence effect magnitude and intervening time between trials.

## 3. Results

Overall summary statistics revealed that observers were highly sensitive to the malignancy discrimination task. Across the user population, sensitivity was 78.72%, specificity was 74.74%, d′ was 1.46, c was −0.065, and the error rate was 18.6%. These metrics indicate that observers were able to perform the dermatological judgment task, consistent with the observers having some degree of expertise. These overall metrics, however, do not reveal whether dermatological judgments on a given image are impacted by sequential dependencies.

Our primary goal was to measure whether serial dependence was present in dermatological judgments. To do this, we calculated the performance metrics above on a trial-wise basis, as a function of the sequential similarity between successive images, as illustrated in Section 2.8.

**Figure 4:**
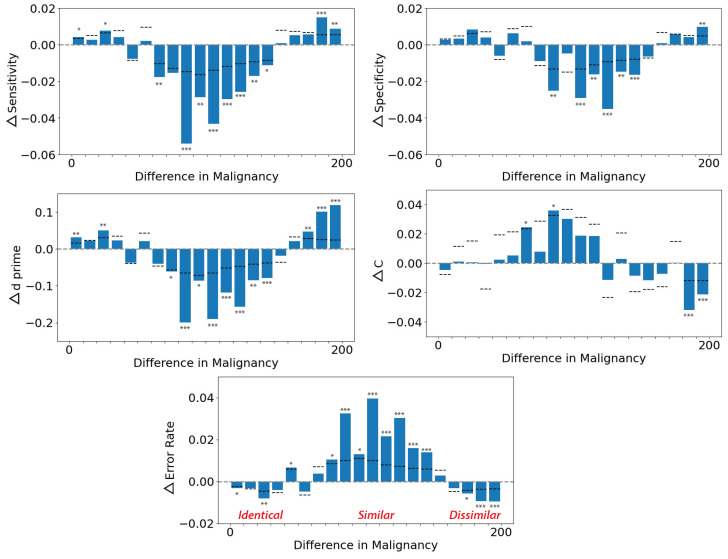
Serial dependence in dermatological classification judgments negatively impacts performance. Performance in the discrimination task was assessed with metrics of sensitivity, specificity, d-prime (d′), criterion (c), and error rate. The abscissa of each graph shows the similarity in the rated malignancy (Figure 2) of successive pairs of images; 0 represents identical successive images, and 200 represents very different sequential images. The ordinate of each graph shows the net change in performance metric (e.g., sensitivity or d′) on the current trial as a function of the similarity of the previous stimulus (N-1 trial) seen by the observer. When the previous stimulus was moderately similar (central regions on the abscissa), all performance metrics dropped, indicating worse performance. For example, when the sequential images were moderately similar, there was an increase in error rates of up to 4.1% on the current trial. Horizontal dashed lines indicate the upper 95% boundary of the permuted null distribution for each bar. Asterisks indicate statistical significance (*:p<0.05;**:p<0.01;***:p<0.001).

Figure 4 shows the net change in sensitivity, specificity, d′, criterion (c), and error rate as a function of the malignancy similarity between current and previous images (1-back or N-1 trial image). The abscissa of each graph shows the similarity in the rated malignancy (Figure 2) of successive pairs of images; 0 represents identical successive images, 200 represents very different sequential images, and the middle range represents similar images. When the previous stimulus was moderately similar (central regions on the abscissa), all performance metrics dropped, indicating worse performance. The worst case occurred when the uncertainty reached the maximum. This is consistent with the findings in previous studies [1,6]. In summary, sensitivity decreased up to 5.4% on the current trial, specificity was decreased up to 3.5% on the current trial, d′ was decreased up to 0.20 on the current trial, criterion (c) was biased up to 0.036 on the current trial, and the error rate was increased up to 4.1% on the current trial. Horizontal dashed lines indicate the upper 95% boundary of the permuted null distribution for each bar. Asterisks indicate statistical significance (p<0.05,0.01,0.001).

**Figure 5:**
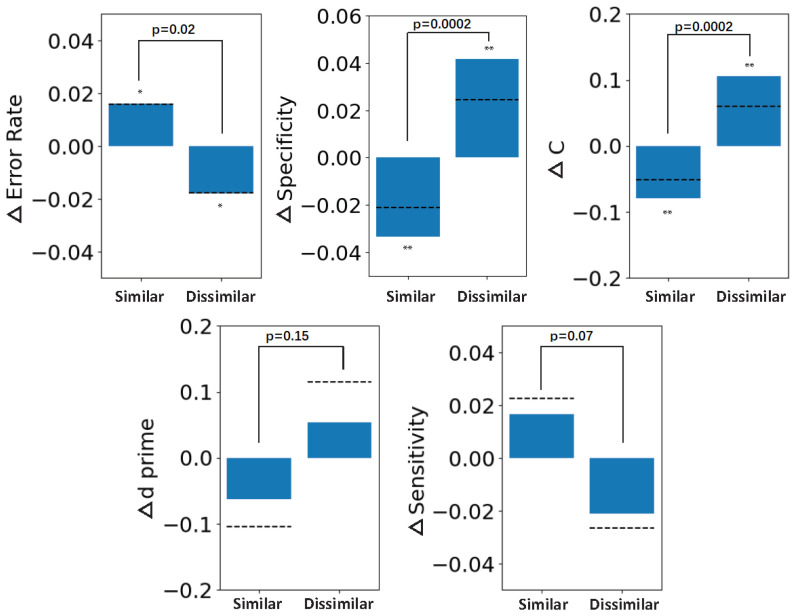
Serial dependence in dermatological discrimination judgments impacts performance. Asterisks indicate statistical significance (*:p<0.05;**:p<0.01). Here, the similarity between sequential images was measured using the LPIPS metric [26]. When similar sequential images were viewed by participants (“similar” on the abscissa), participants had higher error rates, lower specificity, and biased criterion. Sensitivity was not negatively impacted, interestingly, but this was not significant and did not counteract the negative impacts found in all other metrics.

As the semantic similarity via the LPIPS metric is nonlinear, we clustered the performance metrics within small groups into two super-groups, i.e., groups of similar and dissimilar images. The 1-back (N-1) net change in performance for similar and dissimilar sequential images is shown in Figure 5. When similar sequential images were viewed by participants (“similar” on the abscissa), participants had higher error rates, lower specificity, and biased criterion. In particular, the net change in the error rate from similar to dissimilar groups was up to 3.38%, the net change in the specificity from similar to dissimilar groups was up to 7.53%, and the net change in the criterion from similar to dissimilar groups was up to 0.185. The was not a significant change in d′ or sensitivity between similar and dissimilar groups. Overall, there was a negative impact of serial dependence on performance measured by most metrics, including, crucially, the error rate.

**Figure 6:**
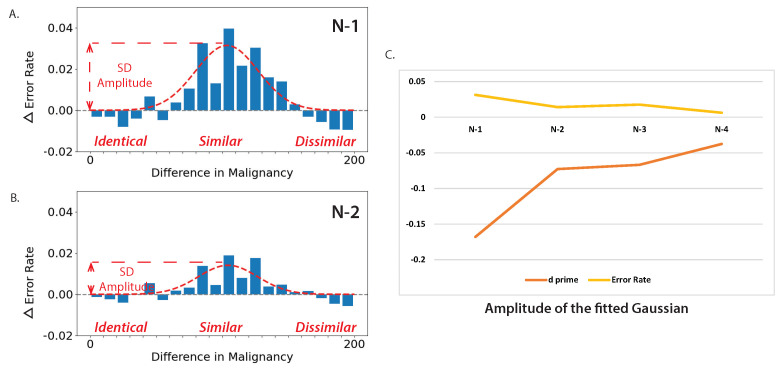
Serial dependence in dermatological discrimination judgments is temporally tuned. (**A**) Error rates such as those in Figure 4 were computed for 1-back trials (just as in Figure 4) and (**B**) for 2-back trials. The increased error rate near the central part of the abscissa indicates that the similarity in the image presented 2 trials before the current trial impacted performance, but less so than the impact of the 1-back stimulus. Gaussian curves were fit to the change in error rates as well as in d′, and the amplitude was taken as a measure of the impact of serial dependence (SD) on error rates and d′. (**C**) The amplitude of the Gaussian—the strength of serial dependence (SD)—was the strongest for the N-1 stimulus and weaker for the following N-2, N-3, and N-4 stimuli, indicating that serial dependence is temporally tuned—stronger for more recent similar stimuli.

After analyzing 1-back (N-1) serial dependence via malignancy similarity, we conducted the same analysis for 2-back (N-2), 3-back (N-3), and 4-back (N-4) trials. Then, Gaussian curves (as described in Equation (1)) were fitted onto the intermediate results of feature tuning as shown in Figure 6A,B. The amplitude was taken as a measure of the impact of serial dependence on error rates and d′. As shown in Figure 6C, the amplitude of the Gaussian was the strongest for the N-1 stimulus and weaker for the following N-2, N-3, and N-4 stimuli, indicating that serial dependence is temporally tuned—stronger for more recent similar stimuli. In particular, the serial dependence (SD) amplitude for error rates decreased from 3.14% to 0.63%, and the SD amplitude for d′ decreased from 0.17 to 0.038.

## 4. Discussion

The goal of this study was to test if there is serial dependence in the perceptual judgments of real skin lesions in a relatively realistic situation akin to remote store-and-forward teledermatology [11,13,32,33]. We found that there was significant serial dependence in observer judgments of malignancy, and this effect was tuned to the similarity in the sequential images. Moreover, the effects were temporally tuned, strongest for more recent similar stimuli, consistent with the diagnostic criteria of serial dependence.

Serial dependence is a specific process in which the brain smooths perceptual interpretations over time to improve efficiency and accuracy and stabilizes the appearance of the natural world [1,2]. Serial dependence has been found in many perceptual tasks ranging from low-level [34] to high-level cognition [35]. It has also been reported in some clinically relevant domains but with less realistic stimuli and tasks [6]. Serial dependence is not a generalized repetition of responses, and it is not just lapsing, central tendency biases, or other artitfacts [1,2,31].

The serial dependence effect we found here is not due to artifacts such as lapsing, central tendency, repeated button presses, or perseverating on the same response. Those kinds of artifacts are problematic, and they can have a serious detrimental influence on dermatological judgments, but they are not serial dependence, per se. As in previous studies [1,23,31], here, we dissociated serial dependence from these other artifacts using three approaches. First, we confirmed that the measured serial dependence effect here was tuned to the sequential similarity between images. A perseverating or stereotyped response (e.g., pressing the same button over and over again for any number of reasons) does not result in biases that are tuned to the similarity between sequential images. Instead, it simply results in a uniform and stable shift in criterion. Second, we dissociated serial dependence from lapsing, stereotyping, and other artifacts by controlling for any biases that seem to depend on the future. Serial dependence is mainly a bias of the current perceptual decision toward past experience. The future stimulus is unpredictable and random, and therefore cannot influence the current decision. However, if there are stereotyped responses (e.g., simply repeating the same button press or central tendency biases), this will result in what seems like the future being predictive of the present. By subtracting out this future bias, we isolated the 1-trial back effect. This approach—measuring and controlling artifacts by using the future—is a common control in studies of serial dependence [6,8,30,31]. Finally, in a third control, we created permuted and shuffled null distributions. These control for overall biases, lapsing, and stereotyped responses among other potential artifacts as well. All of these controls together demonstrate that serial dependence genuinely impacted performance in dermatology judgments.

Previous studies have tried to measure criterion and d′ in dermatological judgments over time [36,37,38], but they did not examine trial-wise effects. Serial dependence is a trial-by-trial effect [1,2,6,8]: sometimes it happens in random sequences (when sequential stimuli are coincidentally similar) and sometimes it does not happen (when sequential stimuli happen to be different). In typical vision science experiments, stimuli are random and their sequential similarity is not measured, considered, or controlled. Serial dependence will therefore not show up in typical analyses because (1) responses are pooled or collapsed across blocks of trials and (2) sequential similarity is unknown or ignored. So, it is not surprising that serial dependence was not found in a previous study [39] because that study did not measure sequential stimulus similarity and it pooled trials together in blocks, washing out any serial dependence that may have been present. The results of the large data set here confirm that serial dependence is likely to be present in other similar data sets, such as [39]. Serial dependence does not show up in simple signal detection metrics such as d′ and criterion unless one takes into account the trial-wise nature of the effect. Serial dependence is not just a shift in the criterion, and it is not just a change in d′. It can result in both shifts in criterion and d′, as we found here, but these are dynamic over time–they fluctuate from trial to trial. We were able to measure changes in SDT (Signal Detection Theory) metrics including d′ and criterion because we analyzed the data in a trial-wise manner and, more importantly, conditioned the analysis on the sequential similarity between stimuli. We found that d′ decreases for similar (non-identical) stimuli. However, if the stimuli are nearly identical or are very different, then there is no decrease in d′. Likewise, we found that criterion changed depending on the sequential similarity between successive stimuli. Both of these results are important: they indicate that standard SDT metrics including d′ and criterion should not be treated as rigid and fixed over time but should be considered as dynamic features that can reflect the fluctuations of stimuli in the world. Future studies of clinician perception and performance should consider the dynamic nature of signal detection metrics.

Serial dependence is a phenomenon that has been observed in many domains, from low-level perception to high-level cognition [1,2,34,35,40]. An outstanding question in the literature on the basic mechanisms of serial dependence is whether feedback might modulate it. For example, one might speculate that trial-wise feedback could reduce or eliminate serial dependence. The results here speak to this question because observers did receive feedback during the task. Despite that feedback, there was still a significant serial dependence that was tuned to both feature similarity and time. This suggests that feedback (even where it is possible) is not a panacea to eliminate serial dependence. Pragmatically, of course, feedback is not possible in clinically relevant settings because there is no prior ground truth in medical image perception. Nevertheless, it is theoretically and practically valuable to know that feedback is not enough to overcome the visual system’s built-in smoothing operations that cause serial dependence.

There are several limitations in this study. First, this study only investigated one source of perceptual bias–serial dependence. Of course, there are other sources of bias, individual observer differences, attentional differences, lapsing, and myriad other sources of error. We controlled these because our goal was to isolate one particular operationally defined source of perceptual bias: serial dependence. Whether there are interactions among serial dependence and other types of perceptual bias is an open question for future research. A second limitation of this study is that the skin cancer images utilized in the experiment contained only two types of lesion, i.e., nevus (benign) and melanoma (malignant). Though the dermatological classification task is similar to realistic skin cancer diagnostic scenarios in some teledermatology settings, it does not fully capture the range or variety of various skin cancer disease types. Moreover, for the images presented to participants, 57.3% were benign and 42.7% were malignant. This deviates from a realistic distribution, where malignant cases are typically much rarer than benign cases. That said, serial dependence does not hinge on the rate of malignancy—it impacts d′ independent of target frequency, and it is, therefore, likely to occur even for rare target situations. However, the issue of disease prevalence remains a very important and open question for future research.

Another limitation is that this study is restricted to store-and-forward teledermatology, which is naturally different from office-based dermatology clinics in several ways, such as available resources and diagnostic procedures. For example, office-based clinicians have multi-modal information about the lesion available, not just photographs, and clinical decisions are more complex than binary ones as in our study. However, during the COVID-19 pandemic, we witnessed a rapid shift from office-based dermatology clinics into teledermatology [41,42]. In line with these recent developments, the teledermatology market size is forecasted to be $67.43 billion in 2030 [43,44]. Accordingly, we chose to investigate remote store-and-forward teledermatology, as it is a highly scalable and increasingly employed form of telemedicine. Finally, it is important to mention that most participants recruited in this study were medical students rather than experts. However, clinicians are not always more accurate than medical students or residents [39]. The reasons for this difference in performance might be the recency of training, attention, or other factors. The simple assumption that trained (older) clinicians are better than less trained (younger) ones is not clear for remote store-and-forward teledermatology, in particular. Future research is needed to explore how expertise might interact with remote teledermatology [39].

There are several additional important avenues of future investigation. Future work should test whether the serial dependence found here is spatially tuned. For example, if sequential images were viewed on different screens (rather than a single mobile device), would there be a reduction in serial dependence? Moreover, how does attention to the task modulate the serial dependence in dermatological judgments? Future studies should address these questions, along with designs that incorporate a larger variety of lesions and a more realistic distribution of malignancy. Finally, future studies should also focus on how to utilize serial dependence tuning functions, i.e., feature tuning and temporal tuning that we found here to potentially alleviate the biases reported here.

## 5. Conclusions

In this study, we analyzed 758,139 skin cancer diagnostic records from an online app in which participants made a series of malignancy discrimination judgments. We quantified sequential malignancy similarity and sequential semantic similarity between successively viewed images, and we investigated classification performance as a function of these similarity metrics. We found significant serial dependence effects in perceptual discrimination judgments, which negatively impacted performance measures, including sensitivity, specificity, and error rates. Moreover, we showed that the serial dependence was tuned to the similarity in the images, and it decayed over time. These findings help understand one potential source of systematic bias and errors in medical image perception tasks and hint at useful approaches that could alleviate the errors due to serial dependence.

## Figures and Tables

**Figure A1:**
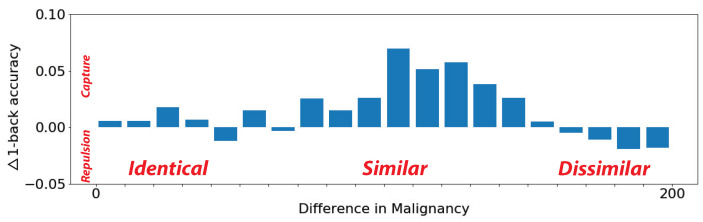
Relationship between difference in malignancy and the 1-back accuracy. The abscissa shows the similarity in the rated malignancy (Figure 2) of successive pairs of images; 0 represents identical successive images, and 200 represents very different sequential images. The ordinate shows the net change in 1-back accuracy on the current trial as a function of the similarity of the previous stimulus (N-1 trial) seen by the observer. When the previous stimulus was moderately similar (central regions on the abscissa), responses were consistently attracted towards the previous stimulus. This pulling effect was up to 7%. The dynamic change of the 1-back accuracy is consistent with performance metrics’ change in Figure 4.

## Data Availability

Not applicable.

## Abbreviations

The following abbreviations are used in this manuscript:

ISIC	The International Skin Imaging Collaboration
GAN	Generative Adversarial Network
LPIPS	Learned Perceptual Image Patch Similarity
AI	Artificial Intelligence
TP	True Positive
FN	False Negative
FP	False Positive
TN	True Negative
FAR	False Alarm Rate
HR	Hit Rate
C	Criterion
d′	d prime
M	Malignancy
I	Image
SDT	Signal Detection Theory
2AFC	Two-Alternative Forced Choice

## Appendix A. Data Preprocessing

In total, 7 of the 13 variables of the data provided by the app are of interest to this research paper and define each data point. They are defined as: User ID (defining a unique ID for each user of the app), score (defining if the answer given has been correct (100) or incorrect (0)), response submitted at (defining at what particular time the response of the user was given), problem appeared at (defining at what particular time the image appeared on the device of the user), origin (defining the image name of the particular image shown), current correct answer (defining if the correct answer is either malignant or benign), and chosen answer (defining if the answer given is either malignant or benign).

Prior to analyses, the following steps were conducted to include only valid data points in the analyses: first, all data points with a larger response time than 3600 s (1 h) were excluded. As data were collected on a smartphone app, it is assumed that for responses over 1 h, the app was running without users paying attention to it. Second, all remaining data points with a longer response time than three standard deviations of the raw data were excluded, which is a common method to exclude outliers [45]. Third, all users with less than 10 trials were excluded to achieve reliable data for the calculation of n-back accuracy. In total, 1083 data points were excluded due to invalidity. The exclusion of these data points did not qualitatively change the pattern of results.

## Appendix B. Evidence of Attracting Effect

Overall, we found significant serial dependence effects in dermatological discrimination judgments. One of the important properties of serial dependence is the attracting effect. Here, we show evidence of attraction in perceptual discrimination judgments as well. We defined the 1-back accuracy as

$$1-\mathrm{back}\;\mathrm{accuracy}=\frac{\#\mathrm{current}\mathrm{response}==\mathrm{previous}\mathrm{stimulus}\mathrm{label}}{\#trials}$$

Next, we measured the net change of the 1-back accuracy relative to what is expected by chance. Similarly, we used the future trial (N + 1) stimulus as the “chance” baseline. If there is an attracting effect, the 1-back accuracy will be greater than 0. In reverse, if a repulsion effect occurs, the 1-back accuracy will be smaller than 0. In summary, we found evidence of an attracting effect when previous stimuli were moderately similar, thus aligning with the serial dependence property (Figure A1).

## References

[1] Fischer J., Whitney D. (2014). Serial dependence in visual perception. Nat. Neurosci..

[2] Cicchini G.M., Mikellidou K., Burr D.C. (2018). The functional role of serial dependence. Proc. R. Soc. B.

[3] Taubert J., Van der Burg E., Alais D. (2016). Love at second sight: Sequential dependence of facial attractiveness in an on-line dating paradigm. Sci. Rep..

[4] Taubert J., Alais D. (2016). Serial dependence in face attractiveness judgements tolerates rotations around the yaw axis but not the roll axis. Vis. Cogn..

[5] Van der Burg E., Toet A., Brouwer A.M., Van Erp J.B. (2021). Serial dependence of emotion within and between stimulus sensory modalities. Multisens. Res..

[6] Manassi M., Ghirardo C., Canas-Bajo T., Ren Z., Prinzmetal W., Whitney D. (2021). Serial dependence in the perceptual judgments of radiologists. Cogn. Res. Princ. Implic..

[7] Ren Z., Stella X.Y., Whitney D. (2022). Controllable medical image generation via GAN. J. Percept. Imaging.

[8] Ren Z., Canas-Bajo T., Ghirardo C., Manassi M., Yu S.X., Whitney D. Serial dependence in perception across naturalistic GAN-generated mammograms.

[9] Perednia D.A., Brown N. (1995). Teledermatology: One application of telemedicine. Bull. Med Libr. Assoc..

[10] Pasquali P., Sonthalia S., Moreno-Ramirez D., Sharma P., Agrawal M., Gupta S., Kumar D., Arora D. (2020). Teledermatology and its current perspective. Indian Dermatol. Online J..

[11] Eedy D., Wootton R. (2001). Teledermatology: A review. Br. J. Dermatol..

[12] Whited J.D. (2006). Teledermatology research review. Int. J. Dermatol..

[13] Warshaw E.M., Hillman Y.J., Greer N.L., Hagel E.M., MacDonald R., Rutks I.R., Wilt T.J. (2011). Teledermatology for diagnosis and management of skin conditions: A systematic review. J. Am. Acad. Dermatol..

[14] Lee J.J., English J.C. (2018). Teledermatology: A review and update. Am. J. Clin. Dermatol..

[15] Hosny K.M., Kassem M.A., Foaud M.M. Skin cancer classification using deep learning and transfer learning. Proceedings of the 2018 9th Cairo International Biomedical Engineering Conference (CIBEC).

[16] Kassem M.A., Hosny K.M., Fouad M.M. (2020). Skin lesions classification into eight classes for ISIC 2019 using deep convolutional neural network and transfer learning. IEEE Access.

[17] Fraiwan M., Faouri E. (2022). On the Automatic Detection and Classification of Skin Cancer Using Deep Transfer Learning. Sensors.

[18] Tensen E., Van Der Heijden J., Jaspers M., Witkamp L. (2016). Two decades of teledermatology: Current status and integration in national healthcare systems. Curr. Dermatol. Rep..

[19] Yim K.M., Florek A.G., Oh D.H., McKoy K., Armstrong A.W. (2018). Teledermatology in the United States: An update in a dynamic era. Telemed. e-Health.

[20] Tschandl P., Rosendahl C., Kittler H. (2018). The HAM10000 dataset, a large collection of multi-source dermatoscopic images of common pigmented skin lesions. Sci. Data.

[21] Codella N.C., Gutman D., Celebi M.E., Helba B., Marchetti M.A., Dusza S.W., Kalloo A., Liopyris K., Mishra N., Kittler H. Skin lesion analysis toward melanoma detection: A challenge at the 2017 international symposium on biomedical imaging (isbi), hosted by the international skin imaging collaboration (isic). Proceedings of the 2018 IEEE 15th International Symposium on Biomedical Imaging (ISBI 2018).

[22] Combalia M., Codella N.C., Rotemberg V., Helba B., Vilaplana V., Reiter O., Carrera C., Barreiro A., Halpern A.C., Puig S. (2019). Bcn20000: Dermoscopic lesions in the wild. arXiv.

[23] Fritsche M., Mostert P., de Lange F.P. (2017). Opposite effects of recent history on perception and decision. Curr. Biol..

[24] Wexler M., Duyck M., Mamassian P. (2015). Persistent states in vision break universality and time invariance. Proc. Natl. Acad. Sci. USA.

[25] Bliss D.P., Sun J.J., d′Esposito M. (2017). Serial dependence is absent at the time of perception but increases in visual working memory. Sci. Rep..

[26] Zhang R., Isola P., Efros A.A., Shechtman E., Wang O. The unreasonable effectiveness of deep features as a perceptual metric. Proceedings of the IEEE Conference on Computer Vision and Pattern Recognition.

[27] Krizhevsky A., Sutskever I., Hinton G.E. (2017). Imagenet classification with deep convolutional neural networks. Commun. ACM.

[28] Simonyan K., Zisserman A. (2014). Very deep convolutional networks for large-scale image recognition. arXiv.

[29] He K., Zhang X., Ren S., Sun J. Deep residual learning for image recognition. Proceedings of the IEEE Conference on Computer Vision and Pattern Recognition.

[30] Maus G.W., Chaney W., Liberman A., Whitney D. (2013). The challenge of measuring long-term positive aftereffects. Curr. Biol..

[31] Pascucci D., Mancuso G., Santandrea E., Della Libera C., Plomp G., Chelazzi L. (2019). Laws of concatenated perception: Vision goes for novelty, decisions for perseverance. PLoS Biol..

[32] Finnane A., Dallest K., Janda M., Soyer H.P. (2017). Teledermatology for the diagnosis and management of skin cancer: A systematic review. JAMA Dermatol..

[33] Villani A., Scalvenzi M., Fabbrocini G. (2020). Teledermatology: A useful tool to fight COVID-19. J. Dermatol. Treat..

[34] Goettker A., Stewart E.E. (2022). Serial dependence for oculomotor control depends on early sensory signals. Curr. Biol..

[35] Manassi M., Whitney D. (2022). Illusion of visual stability through active perceptual serial dependence. Sci. Adv..

[36] Warshaw E.M., Lederle F.A., Grill J.P., Gravely A.A., Bangerter A.K., Fortier L.A., Bohjanen K.A., Chen K., Lee P.K., Rabinovitz H.S. (2009). Accuracy of teledermatology for pigmented neoplasms. J. Am. Acad. Dermatol..

[37] Lamel S.A., Haldeman K.M., Ely H., Kovarik C.L., Pak H., Armstrong A.W. (2012). Application of mobile teledermatology for skin cancer screening. J. Am. Acad. Dermatol..

[38] Wang R.H., Barbieri J.S., Nguyen H.P., Stavert R., Forman H.P., Bolognia J.L., Kovarik C.L. (2020). Clinical effectiveness and cost-effectiveness of teledermatology: Where are we now, and what are the barriers to adoption?. J. Am. Acad. Dermatol..

[39] Wolfe J.M. (2022). How one block of trials influences the next: Persistent effects of disease prevalence and feedback on decisions about images of skin lesions in a large online study. Cogn. Res. Princ. Implic..

[40] Kiyonaga A., Scimeca J.M., Bliss D.P., Whitney D. (2017). Serial dependence across perception, attention, and memory. Trends Cogn. Sci..

[41] Perkins S., Cohen J.M., Nelson C.A., Bunick C.G. (2020). Teledermatology in the era of COVID-19: Experience of an academic department of dermatology. J. Am. Acad. Dermatol..

[42] Price K.N., Thiede R., Shi V.Y., Curiel-Lewandrowski C. (2020). Strategic dermatology clinical operations during the coronavirus disease 2019 (COVID-19) pandemic. J. Am. Acad. Dermatol..

[43] Insights F.B. (2020). Teledermatology Market Size, Share & COVID-19 Impact Analysis, and Regional Forecast 2021–2028. https://www.fortunebusinessinsights.com/teledermatology-market-103491.

[44] Research P. (2021). Teledermatology Market—Global Industry Analysis, Size, Share, Growth, Trends, Regional Outlook, and Forecast 2021–2030. https://www.precedenceresearch.com/teledermatology-market.

[45] Miller J. (1991). Reaction time analysis with outlier exclusion: Bias varies with sample size. Q. J. Exp. Psychol..

